# Switch Tandem Repeats Influence the Choice of the Alternative End-Joining Pathway in Immunoglobulin Class Switch Recombination

**DOI:** 10.3389/fimmu.2022.870933

**Published:** 2022-05-16

**Authors:** Chloé Oudinet, Xuefei Zhang, Nadine Puget, Nia Kyritsis, Claire Leduc, Fatima-Zohra Braikia, Audrey Dauba, Frederick W. Alt, Ahmed Amine Khamlichi

**Affiliations:** ^1^Institut de Pharmacologie et de Biologie Structurale, IPBS, Université de Toulouse, CNRS, Université Paul Sabatier, Toulouse, France; ^2^Program in Cellular and Molecular Medicine, Howard Hughes Medical Institute, Department of Genetics, Boston Children’s Hospital, Harvard Medical School, Boston, MA, United States

**Keywords:** B lymphocyte, class switch recombination, switch sequence, alternative end-joining, enhancer

## Abstract

Immunoglobulin class switch recombination (CSR) plays an important role in humoral imm\une responses by changing the effector functions of antibodies. CSR occurs between highly repetitive switch (S) sequences located upstream of immunoglobulin constant gene exons. Switch sequences differ in size, the nature of their repeats, and the density of the motifs targeted by the activation-induced cytidine deaminase (AID), the enzyme that initiates CSR. CSR involves double-strand breaks (DSBs) at the universal Sµ donor region and one of the acceptor S regions. The DSBs ends are fused by the classical non-homologous end-joining (C-NHEJ) and the alternative-NHEJ (A-NHEJ) pathways. Of the two pathways, the A-NHEJ displays a bias towards longer junctional micro-homologies (MHs). The Sµ region displays features that distinguish it from other S regions, but the molecular basis of Sµ specificity is ill-understood. We used a mouse line in which the downstream Sγ3 region was put under the control of the Eµ enhancer, which regulates Sµ, and analyzed its recombination activity by CSR-HTGTS. Here, we show that provision of Eµ enhancer to Sγ3 is sufficient to confer the recombinational features of Sµ to Sγ3, including efficient AID recruitment, enhanced internal deletions and robust donor function in CSR. Moreover, junctions involving Sγ3 display a bias for longer MH irrespective of sequence homology with switch acceptor sites. The data suggest that the propensity for increased MH usage is an intrinsic property of Sγ3 sequence, and that the tandem repeats of the donor site influence the choice of the A-NHEJ.

## Introduction

Developing B lymphocytes remodel the variable regions of their immunoglobulin (Ig) loci through V(D)J recombination, generating a vast array of antigenic specificities (1–3). Upon antigen encounter, mature B lymphocytes undergo class switch recombination (CSR) which targets the constant (*C_H_*) genes of the *Ig* heavy chain (*IgH*) locus, ultimately leading to a change of the constant domain of Ig molecules. CSR thus enables activated B cells to switch from the expression of the initial IgM to the expression of downstream isotypes (IgG, IgE or IgA) with novel effector functions (4–7). CSR occurs between highly repetitive, GC-rich, switch (S) sequences, located upstream of the *C_H_* gene exons, except *Cδ*. CSR to a particular S region is induced by specific external stimuli including antigens, mitogens, cytokines, and inter-cellular interactions, and requires transcription across S regions, directed by the so-called I promoters (4–7).

Switch transcription is regulated by various long-range *cis*-acting elements, including enhancers and insulators (8, 9). The major control element is a super-enhancer called 3’ Regulatory Region (3’RR), composed of four enhancers that act in synergy to activate upstream I promoters (8, 9). Regulation of I promoters involves dynamic conformational changes that are controlled in a developmental stage-, and stimulus-dependent manner (9). In resting B cells, the 3’RR engages in stable interactions with Eµ enhancer, located upstream of *C_H_* genes (10), forming a CSR centre (CSRC) (11). Upon activation, the primed I promoter is brought to the CSRC where synapsis between Sµ and the partner switch sequence is promoted by Cohesin-mediated loop extrusion that is dynamically impeded by Eµ enhancer and the 3’RR ( (11). Reviewed in Ref (9)).

Switch transcription generates long non-coding RNAs that promote accessibility of S sequences, through secondary structures such as R loops and G quadruplexes (12–15), to the enzyme Activation-Induced cytidine Deaminase (AID), which is absolutely required for CSR (16, 17). AID initiates the process by deaminating cytosines to uracils (5, 18). Processing of uracils by base excision and mismatch repair pathways leads to double-strand break (DSB) intermediates, at the Sµ donor region and one of the acceptor S regions (Sγ, Sε, Sα). The DSBs are monitored by components of the DNA damage response pathway (such as ATM, 53BP1 and H2AX) and repaired by the classical and alternative non-homologous end joining pathways (hereafter C-NHEJ and A-NHEJ, respectively) (19–25), ultimately fusing Sµ and the acceptor S region, with a strong bias towards deletional joining (26). C-NHEJ and A-NHEJ use different components and have different signatures at switch junctions. The C-NHEJ pathway (whose core components include Ku70, Ku80, XRCC4, and ligase 4) favors blunt ends or ends with limited MH (≤3 bp), and is the major repair pathway in CSR (27, 28). The A-NHEJ pathway uses components such as CtIP, MRN, and PARP-1, favors ends with larger MHs (≥4 bp) and involves extensive end resection (27–30).

The S regions differ in size, ranging from _~_2 kb (Sε) to _~_12 kb (Sγ1), and there is evidence that the number of tandem repeats determines, at least in part, the efficiency of CSR (31, 32). They also differ in the nature of their tandem repeats (4, 33). Sµ, Sε and Sα core repeats are short (5 bp), consisting of units such as GAGCT and GGGG/CT, whereas the Sγ (Sγ3, Sγ1, Sγ2b and Sγ2a in the mouse) core repeats, which also contain GAGCT and GGGG/CT units, are longer (48-49 bp) and more complex (4–6, 34). Sµ has a higher sequence homology with Sα and Sε than with Sγ. Likewise, Sγ3 sequence for instance displays higher sequence homology with the other Sγ than with Sµ, Sε and Sα (4, 28, 34). In this regard, it was suggested that A-NHEJ could play an important role in CSR involving S partners with substantial sequence homology (22, 25, 35). However, the extent to which S core repeats’ peculiarities influence their CSR efficiency and the choice of the NHEJ pathway is still unclear.

Various studies revealed that Sµ region displays specific features that distinguish it from downstream S regions. For instance, Sµ is transcribed along B cell development, whereas other S regions are mainly transcribed in activated mature B cells (9). Additionally, Sµ is the most repetitive and displays the highest density of AID target motifs (4), in particular of the evolutionary conserved AGCT motif (36) (see **Supplementary Table 1**). Following activation for CSR, internal switch deletions (ISDs) are detected at Sµ region at a higher frequency than at downstream S regions [*e.g *(35, 37–41)] Moreover, mice deficient for components of the DNA damage response or C-NHEJ feature defects in CSR but not in Sµ ISDs (35, 39–42), suggesting that DNA repair mechanisms involved in ISDs differ, at least in part, from those involved in genuine CSR [discussed in (27)]. Several non-mutually exclusive hypotheses could be put forward to account for Sµ specificity including Eµ enhancer proximity, continuous transcription, chromatin structure, preferential recruitment of AID, and differential recruitment of repair pathways. Thus, the molecular basis of Sµ specificity remains elusive.

We reasoned that by putting a downstream S sequence under the control of the known elements that regulate Sµ, we could investigate if that S region can acquire Sµ properties. To this end, we used a mouse line in which Iγ3 promoter was replaced by a pre-rearranged VDJ-Eµ cassette (43), leaving intact the endogenous Sµ and Sγ3 regions. In this setting, the two S regions have roughly the same size, are almost equally distant from Eµ enhancer, but differ in the nature of their core repeats and the density of AID target motifs. Here, we focused on the recombinational activity of the two S regions. We show that Sγ3 acquired most of Sµ properties but displayed a distinctive propensity for longer MH in both ISDs and CSR.

## Materials and Methods

### Mice and Ethical Guidelines

The WT and mutant mice are of 129Sv background. All analyses were performed on homozygous A150^Δ/Δ^ or A150^Δ/Δ^ AID^-/-^ mice. 6-8 weeks-old mice were used. All experiments on mice have been carried out according to the CNRS ethical guidelines and were approved by the Regional Ethical Committee (Accreditation N° E31555005).

### Generation of A150 Mice

Mice were generated as previously described (43).

### Antibodies and Cytokines

FITC-conjugated anti-IgG3 and anti-IgA antibodies were purchased from BD-Pharmingen. APC-conjugated anti-B220, PE-conjugated anti-IgM, FITC-conjugated anti-IgG1, IL4, TGF-β, BLyS, and IL5 were from BioLegend. LPS was purchased from Sigma, anti-IgD-dextran from Fina Biosolutions, and anti-CD40 from eBiosciences. Anti-IgG antibody was purchased from Diagenode and anti-AID antibody from Abcam.

### Splenic B-Cell Activation

Single cell suspensions from spleens were obtained by standard techniques and splenic B cells were negatively sorted using CD43-magnetic microbeads and LS columns (Miltenyi). To induce switch transcription and CSR, negatively sorted CD43^-^ splenic B cells were cultured for 2 days and 4.5 days, respectively, at a density of 5 × 10^5^ cells per ml in the presence of LPS (25 µg/ml) + anti-IgD-dextran (3 ng/ml) (hereafter LPS stimulation), LPS (25 µg/ml) + anti-IgD-dextran (3 ng/ml) + IL4 (25 ng/ml) (LPS+IL4 stimulation), anti-CD40 (1 µg/ml) + IL4 (25 ng/ml) (anti-CD40+IL4 stimulation), or anti-CD40 (1 µg/ml) + IL4 (10 ng/ml) + IL5 (5 ng/ml) + BLyS (5 ng/ml) + TGF-β (2 ng/ml) (anti-CD40+TGF-β stimulation).

### Fluorescence-Activated Cell Sorting (FACS) Analyses

Single-cell suspensions from spleens from 6- to 8-weeks old mice were prepared by standard techniques. Cells (1 × 10^6^ cells/assay) were stained and gated as indicated in figure legends. Data on 1 × 10^4^ viable cells were obtained using a BD LSR Fortessa X-20 flow cytometer.

### Primers

All the primers used in this study are listed in the **Supplementary Table 2**.

### Reverse Transcription-qPCR (RT-qPCR)

Total RNAs were prepared from WT and A150 splenic B cells at d2 post-stimulation, reverse transcribed (Invitrogen) and subjected to qPCR using Sso Fast Eva Green (BioRad). *Actin* transcripts were used for normalization and the results are shown as percentage of actin. The primers used have been described (44).

### Chromatin Immunoprecipitation (ChIP)

Chromatin was prepared from 5 × 10^6^ d2-activated splenic B cells. Chromatin was cross-linked for 10 min at RT with 1% formaldehyde, followed by quenching with 0.125 M glycine. Cross-linked chromatin was then lysed (0.5% SDS, 50 mM Tris, 10 mM EDTA, 1× PIC) and sonicated for 20 cycles 30 s ON–30 s OFF (Diagenode Bioruptor). Sonicated chromatin was diluted 10 times (0.01% SDS, 1.1% Triton X-100, 1.2 mM EDTA, 16.7 mM Tris–HCl, 167 mM NaCl) and precleared with 100 μl of Dynabeads protein-A magnetic beads (Invitrogen) and 5 μl of anti-IgG (Diagenode) for 2 h at 4°C. 5-10% of the precleared chromatin was used as the input sample. Immunoprecipitations were performed overnight at 4°C with 1 × 10^6^ cells and 0.5 μg of anti-AID (Abcam, ab59361) or control anti-IgG (Diagenode, C15410206) per immunoprecipitation. Immunoprecipitated material was recovered with protein A magnetic beads (2 h at 4°C) and washed. Crosslinking was reversed overnight at 45°C. Eluted DNA was extracted by standard techniques and subjected to qPCR. Results are presented as fold enrichment, taking into account both the input and the negative (IgG) sample.

### CSR-HTGTS-Seq

Genomic DNAs were purified from day4-anti-CD40+IL4-activated WT and A150 splenic B cells and were processed exactly as previously described (45). Specific baits were designed upstream of Sμ and Sγ3 regions in A150 mice that distinguish CSR events involving each S region.

### Statistics

Results are expressed as mean ± SD, and overall differences between values were evaluated by an unpaired two-tailed *t* test. ns, not significant, * *p* < 0.05, ** *p* < 0.005, *** *p* < 0.0005, **** *p* < 0.0001.

## Results

We have previously shown that replacement of Iγ3 switch promoter by a PV_H_-VDJ-Eµ cassette (hereafter A150 mutation or mouse line) (**Supplementary Figure 1A**) leads to an accumulation of partially rearranged DJ_H_ alleles and a drastic reduction of V_H_-DJ_H_ recombination (43). Consequently, IgM expression is severely impaired in A150 homozygous mice and B cell development is driven by IgG3 (43) (and **Supplementary Figure 1B**). In this study, we used this mouse line to investigate if Sγ3 region, in its new setting, has acquired the recombinational properties of Sµ.

### Switch Transcription and CSR in A150 B Cells

In normal B cells, Sµ transcription driven by Eµ/Iµ enhancer/promoter is constitutive (46, 47), whereas transcription of downstream S regions driven by their I promoters is inducible (5, 6, 9). In A150 B cells, Sγ3 transcription is driven by the ectopic Eµ/Iµ enhancer/promoter (**Supplementary Figure 2A**). This raises two questions: 1) is Sγ3 constitutively transcribed? and 2) does this setting impact the constitutive transcription of Sµ? We found comparable levels of Sµ transcripts in WT and A150 resting B cells (**Supplementary Figures 2A, B**), and between Sµ and Sγ3 transcripts in A150 resting B cells (**Supplementary Figures 2A, C**). These data suggest that in resting mutant B cells, Sγ3 transcription has become constitutive and does not alter Sµ transcript levels.

It is well established that switch transcription is absolutely required for CSR. To investigate how the mutation affects switch transcription and CSR in activated A150 B cells, sorted CD43^-^ splenic B cells were cultured in the presence of anti-CD40+IL4 (which induces Sγ1 and Sε transcription and CSR to IgG1 and IgE) or with anti-CD40+TGF-β (which induces Sα transcription and CSR to IgA). At day 2 post-stimulation, switch transcript levels were quantified by RT-qPCR. We found a moderate increase of Sµ transcript levels in both anti-CD40+IL4- and anti-CD40+TGF-β-activated A150 B cells compared to WT controls (**Supplementary Figures 3A, B**), and Sµ transcript levels appeared to be slightly higher than Sγ3 in activated A150 B cells under both stimulation conditions (**Supplementary Figure 3A, C**). With respect to downstream isotypes, Sγ1, transcript levels were slightly reduced in activated A150 B cells, whereas Sε and Sα transcript levels were unaffected (**Supplementary Figure 3D**).

To investigate the impact of the replacement mutation on CSR, surface Ig (sIg) expression was monitored by FACS at day 4.5 post-stimulation. Both sIgG1 and sIgA were reduced following appropriate stimulation of A150 B cells (**Supplementary Figure 4**). sIgE expression was not assayed upon anti-CD40+IL4 stimulation as non-specific staining is caused by soluble IgE binding to FcεRII expressed by activated B cells. Thus, the replacement mutation leads to reduced surface expression of IgG1 and IgA (see *Discussion*).

### Efficient Recruitment of AID by Sγ3 Region in Activated A150 B Cells

Switch transcription is mechanistically important for AID targeting to S regions (7, 48). Analysis of switch transcription revealed that Sγ3 in activated A150 B cells was robustly transcribed, though slightly less than Sµ (**Supplementary Figure 3C**). We thus asked if Sγ3 region could act as a switch donor site. As a first approach, we performed a ChIP-qPCR assay to detect potential enrichment of AID at Sγ3 region in two stimulation conditions: LPS and LPS+IL4.

**Figure 1:**
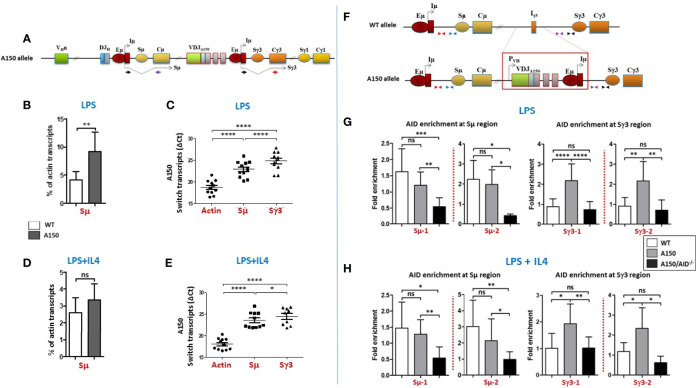
Switch transcription and AID recruitment at Sµ and Sγ3 regions. **(A)** The top scheme indicates the structure of the A150 allele and Sµ and Sγ3 transcripts each derived from its proximal Eµ/Iµ enhancer/promoter. The relative position of the primers used to detect spliced switch transcripts is indicated. **(B)** Quantification of Sµ transcript levels in LPS-activated WT and A150 B cells. Total RNAs were prepared from purified CD43^-^ WT and A150 B cells at day 2 post-stimulation, reverse transcribed, and Sµ transcript levels quantified by RT-qPCR (n = 4). **(C)** Comparison of Sµ and Sγ3 transcript levels in LPS-activated A150 B cells. Quantification of switch transcript levels was as in **(B)**. Because the Cµ and Cγ3 reverse primers are different, the ΔCt data are shown (n = 4). **(D)** Quantification of Sµ transcript levels in LPS+IL4-activated WT and A150 B cells. Quantification of Sµ transcript levels was as in **(B)** (n = 4). **(E)** Comparison of Sµ and Sγ3 transcript levels in LPS+IL4-activated A150 B cells. Quantification of switch transcript levels was as in **(C)** (n = 4). **(F)** The top scheme indicates the relative position of the primers used for qPCR. **(G, H)** A150 Sγ3 region efficiently recruits AID. AID recruitment was assayed at two similarly distant sites upstream of Sµ and Sγ3 regions by analytical ChIP-qPCR. The assays were performed on chromatin from activated B cells of the indicated genotypes at day 2 post-stimulation with LPS **(G)** or LPS+IL4 **(H)** A150: homozygous for A150 mutation, A150/AID^-/-^: double-homozygous mutant (for both A150 and AID) (n = 4). ns, not significant, *p < 0.05, **p < 0.005, ***p < 0.0005, ****p < 0.0001.

We first quantified Sµ and Sγ3 transcript levels, and found that A150 Sµ transcript levels were higher than their WT counterparts upon LPS stimulation (**Figures 1A, B**), while they were comparable following LPS+IL4 stimulation (**Figure 1D**). In both stimulation conditions, A150 Sµ transcript levels were relatively higher than A150 Sγ3 levels (**Figures 1C, E**). On the other hand, Sγ3 transcripts levels were comparable between WT and A150 B cells upon LPS stimulation (**Supplementary Figures 5A, B**).

We assayed for AID recruitment at two similarly distant sites upstream of Sµ and Sγ3 regions (**Figure 1F**). As a negative control, we used chromatin derived from activated AID-deficient A150 B cells.

The data show that AID was enriched at Sµ region of both WT and A150 B cells, regardless of the stimulation condition (**Figures 1G, H**). In contrast, while AID recruitment was at the background level at Sγ3 region in WT B cells, it was readily detected at Sγ3 in A150 B cells in both stimulation conditions (**Figures 1G, H**). Altogether, the data suggest that Sγ3 region efficiently recruits AID in activated A150 B cells.

### Sγ3 Can Act as a Powerful Switch Donor Site in Activated A150 B Cells

**Figure 2:**
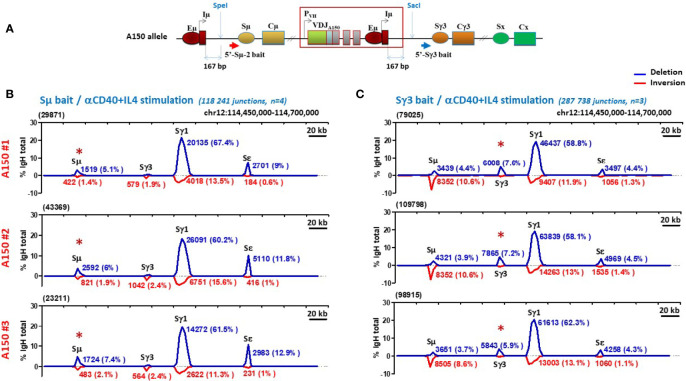
Sγ3 under the control of Eµ enhancer acts as a powerful switch donor site. **(A)** The scheme shows the A150 allele with the relative position with respect to Iµ exon of the Sµ and Sγ3 baits used in CSR-HTGTS assays. **(B, C)** CSR-HTGTS assays were performed on CD43^-^ sorted A150 splenic B cells induced to switch with anti-CD40+IL4 (aCD40+IL4). At day 4.5 post-stimulation, genomic DNAs were purified and subjected to CSR-HTGTS. CSR-HTGS analyses measure joining of the 5′ end of DSBs in the 5′ regions of either Sμ (Sµ bait) **(B)**, or Sγ3 (Sγ3 bait) **(C)** to the other S regions, involving either deletions (blue curves) or inversions (red curves). For each isotype, the number of junction sequences and the corresponding percentages are indicated on the top of the curves. The total number of switch junctions and of independent mice are indicated between brackets, together with the stimulation condition. The red asterisk on the top of Sµ and Sγ3 indicates the location of the bait upstream of Sµ and Sγ3 respectively.

In order to directly explore if Sγ3 can act as a switch donor site, we performed CSR-high throughput genome-wide translocation sequencing (CSR-HTGTS) (45) which provides a comprehensive view of the recombination events at the genomic level. The assay was performed on A150 B cells activated with anti-CD40+IL4 by using primers specific of Sµ and Sγ3 regions as baits (**Figure 2A**). Analysis of tens of thousands of junction sequences revealed several interesting features.

With regard to the deletional events, by using Sµ primer as a bait, _~_6% of joins corresponded to ISD joins within A150 Sµ region. As expected, the majority (_~_63%) corresponded to CSR Sµ/Sγ1 joins, and only _~_11% to Sµ/Sε joins (**Figure 2B**, blue curves). A similar profile was seen when a Sγ3 primer was used as a bait: _~_7% of joins corresponded to Sγ3 ISD joins, the majority (_~_60%) corresponded to CSR Sγ3/Sγ1 joins, and only _~_4.5% to Sγ3/Sε joins (**Figure 2C**, blue curves).

With respect to the inversional events, the Sµ bait detected low levels of inversions in the context of Sµ ISDs (_~_1.8%), and _~_2.2% of Sµ/Sγ3 and _~_0.8% of Sµ/Sε inversions in the context of CSR. Sµ/Sγ1 inversions were more frequent (_~_13.5%) (**Figure 2B**, red curves). The Sγ3 bait did not detect inversions within Sγ3 ISDs. In contrast, _~_10% of Sγ3/Sµ, _~_13% of Sγ3/Sγ1, and _~_1.3% of Sγ3/Sε joins were inversions (**Figure 2C**, red curves).

**Figure 3:**
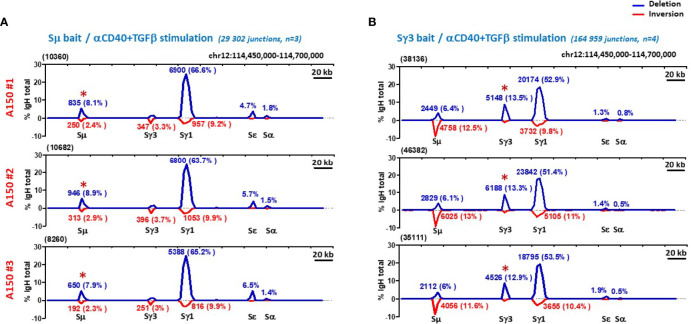
Sγ3 under the control of Eµ enhancer acts as a powerful switch donor site. **(A, B)** CSR-HTGTS assays were performed on CD43^-^ sorted WT splenic B cells induced to switch with aCD40+TGF-β, and analyzed as in **Figure 2**.

To exclude that the acquired capacity of Sγ3 to function as a strong donor is stimulus-dependent, we repeated the same assay following induction of CSR with anti-CD40+TGF-β. We found that globally, A150 Sγ3 acted as a robust switch donor site in this stimulation condition (**Figures 3A, B**).

By focusing on the donor function of Sµ and Sγ3 irrespective of the acceptor site and the orientation of recombination events (thus leaving aside Sµ and Sγ3 ISDs), the overall efficiency and switching pattern of Sγ3 was roughly similar to that of Sµ (**Figures 2B, C**, and **3A, B**).

Together, the data show that in activated A150 B cells, Sγ3 acts as a powerful switch donor site and undergoes internal deletions with comparable efficiency to Sµ.

### Increased Micro-Homology Usage by Sγ3 During Genuine CSR

It is generally assumed that blunt ends or ends with limited MH (≤3 bp) are the preferential substrates of C-NHEJ, whereas ends with longer MH (≥4 bp) involve the A-NHEJ preferentially (27, 28). Having shown that Sγ3 can act as a robust donor site, we asked to what extent the nature of Sγ3 repeats impacts the pattern of switch junctions. We addressed this question by comparing junction sequences involving partner S sequences with high or low sequence homology to Sγ3, following either anti-CD40+IL4 or anti-CD40+TGF-β stimulation.

**Figure 4:**
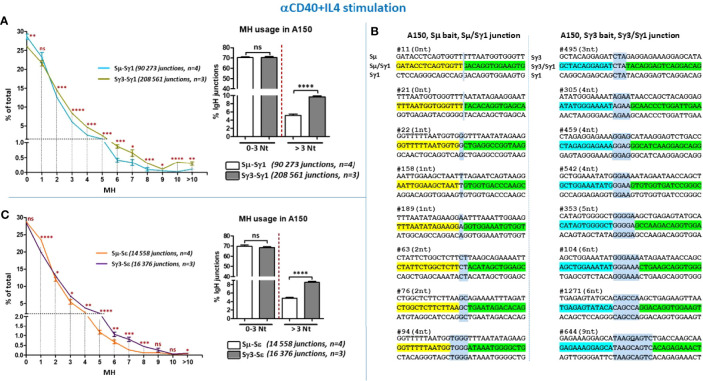
Increased micro-homology usage in switch junctions involving Sγ3 as a donor site. **(A)** MH-mediated joining was analyzed in A150 B cells stimulated with aCD40+IL4 for 4.5 days. MH usage from junctions with blunt and up to 3-bp MH (fused in the right panel), and >3-bp MH were plotted as percentage of total junctions involving Sµ or Sγ3 as switch donor sites and Sγ1 as acceptor site. **(B)** Examples of switch junctions obtained with either Sµ (left panel) or Sγ3 (right panel) as donor sites and Sγ1 as acceptor site. MH at switch junctions is highlighted in pale blue box. **(C)** MH usage from junctions with blunt and up to 3-bp MH (fused in the right panel), and >3-bp MH were plotted as percentage of total junctions involving Sµ or Sγ3 as switch donor sites and Sε as acceptor site. The number of switch junctions and of independent mice are indicated between brackets. The *p* values were calculated by unpaired two-tailed *t* test. ns, not significant, *p < 0.05, **p < 0.005, ***p < 0.0005, ****p < 0.0001.

The data show that the percentage of Sµ/Sγ1 CSR junctions with direct joins was slightly higher than for Sγ3/Sγ1 (**Figure 4A**, left panel, **Figure 4B**, and **Supplementary Figure 6A**). Junctions with 1 bp MH were comparable between Sµ/Sγ1 and Sγ3/Sγ1 (**Figure 4A**, left panel). In contrast, with increased MH, starting from 2 bp MH, Sγ3/Sγ1 joins were consistently more frequent than Sµ/Sγ1 joins (**Figure 4A**, left panel). Taking into account direct joins and 1-3 bp MH (reflecting C-NHEJ involvement), the percentages of Sµ/Sγ1 and Sγ3/Sγ1 joins were comparable, whereas MH > 3 bp (reflecting A-NHEJ involvement) was consistently more frequent in joins involving Sγ3 as a donor (**Figure 4A**, left and right panels, **Figure 4B**, and **Supplementary Figure 6A**). An overall similar profile was found for CSR junctions involving Sε in anti-CD40+IL4-activated A150 B cells (**Figure 4C**, and **Supplementary Figures 6B, C**).

**Figure 5:**
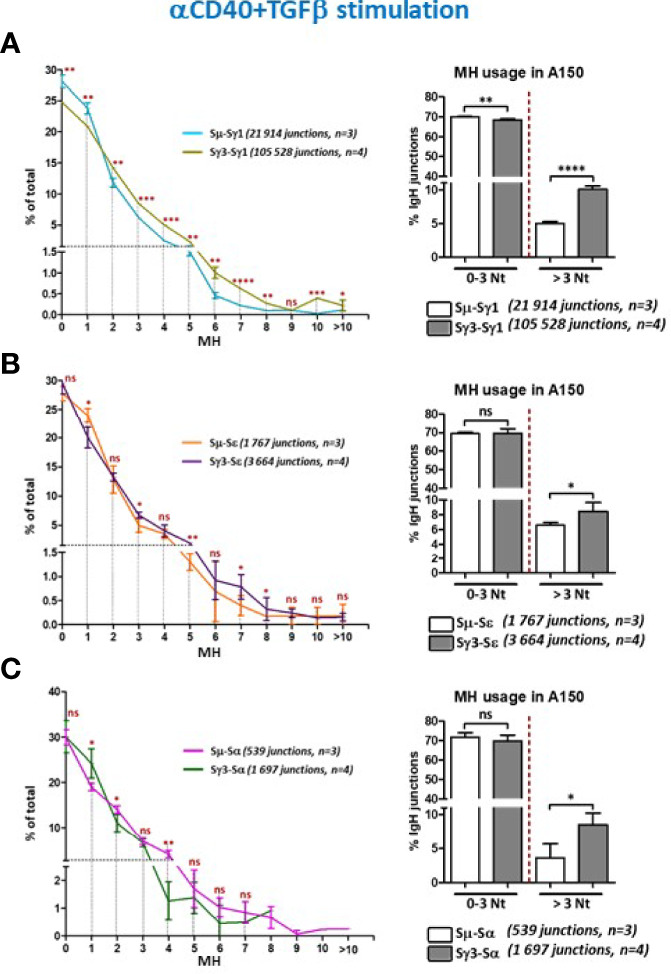
Increased micro-homology usage in switch junctions involving Sγ3 as a donor site. **(A–C) ** MH-mediated joining was analyzed in A150 B cells stimulated with aCD40+TGF-β for 4.5 days. MH usage from junctions with blunt and up to 3-bp MH (fused in the right panels), and >3-bp MH were plotted as percentage of total junctions involving Sµ or Sγ3 as switch donor sites and either Sγ1 **(A)**, Sε **(B)**, or Sα **(C)** as acceptor sites. The number of switch junctions and of independent mice are indicated. The *p* values were calculated by unpaired two-tailed *t* test. ns, not significant, *p < 0.05, **p <0.005, ***p < 0.0005, ****p < 0.0001.

When we assayed for CSR junctions in anti-CD40+TGF-β-activated B cells (**Figures 5A**–**C**, and **Supplementary Figures 7A**–**D**), the level of MH at switch junctions globally resembled that seen with anti-CD40+IL4 stimulation. We note a slight divergence from this pattern for CSR events involving Sε and Sα junctions in anti-CD40+TGF-β-activated B cells (**Figures 5B, C**, left panels), likely due to the low number of junction sequences collected. Nonetheless, the general trend is similar.

Overall, switch junctions displaying more than 3 bp MH were _~_2 times more frequent when Sγ3 was the donor site, irrespective of the stimulation condition or the acceptor site, *i.e.* with higher sequence homology (Sγ1) or lower homology (Sε and Sα) (**Figures 4**, **5**, and **Supplementary Figures 6A, B** and **7A**–**C**).

Thus, the recombination activity of Sγ3 as a donor site leads to increased MH usage regardless of the identity of the acceptor site or the stimulation condition.

### Increased Micro-Homology Usage by Sγ3 During Internal Switch Deletions

The finding of normal ISDs despite decreased CSR in B cells deficient for DNA damage response or C-NHEJ suggested the involvement of different repair mechanisms (35, 39–42). In particular, it was proposed that the repetitiveness of individual S regions and the short-range joining in ISDs may provide more MH and favor A-NHEJ than the long-range joining of different S regions which favors C-NHEJ [discussed in (27)]. This context is also different from *bona fide* CSR where properties of acceptor S regions can potentially influence the choice of the repair pathway. ISDs were previously detected by Southern blot on genomic DNAs derived from IgM^+^ B cell hybridomas, which is not sensitive enough to detect small deletions and may therefore underestimate the frequency of ISDs (27), this is not the case with CSR-HTGTS.

As mentioned, Sµ and Sγ3 differ in the nature of their repeats but undergo an apparently similar frequency of ISDs in activated A150 B cells. This enabled us to investigate the impact of the repeats of each S region on the choice of A-NHEJ *versus* C-NHEJ in short range joining.

**Figure 6:**
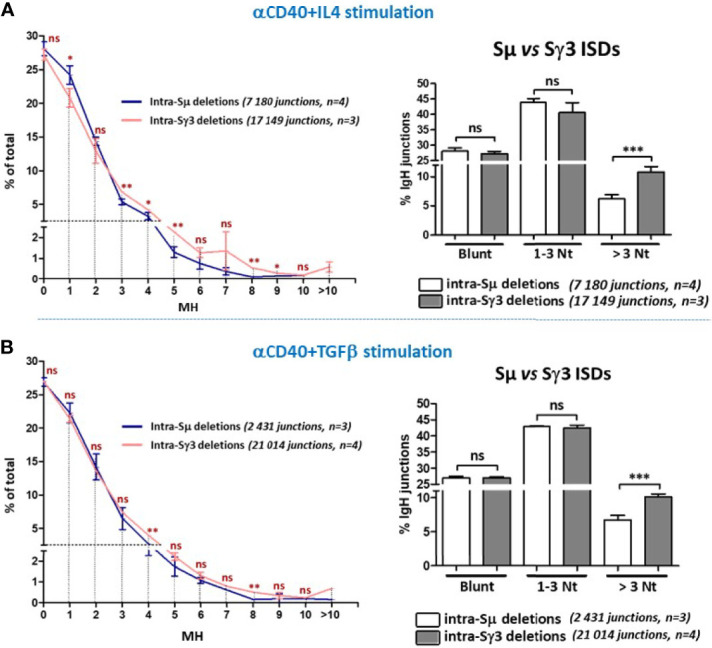
Increased micro-homology usage in Sγ3 internal deletions. MH-mediated joining was analyzed in A150 B cells stimulated with aCD40+IL4 or aCD40+TGF-β for 4.5 days, as in **Figures 4**, **5**. MH usage from junctions with blunt and up to 3-bp MH are displayed separately in the right panels. The *p* values were calculated by unpaired two-tailed *t* test. ns, not significant, *p < 0.05, **p < 0.005, ***p < 0.0005.

The data show that direct joins or junctions with limited MH (1-3 bp) occur at comparable frequencies in Sµ and Sγ3 ISDs following both anti-CD40+IL4 (**Figure 6A**, left and right panels) and anti-CD40+TGF-β stimulation (**Figure 6B**, left and right panels). For both Sµ and Sγ3 ISDs, the C-NHEJ (0-3 MH) remains the most prominent repair pathway (**Figures 6A** **, B**, left and right panels). In contrast, Sγ3 ISDs displayed increased MH usage irrespective of the stimulation condition (**Figures 6A, B**, left and right panels), indicating a more frequent recruitment of the A-NHEJ pathway.

The data strongly suggest that the propensity to use longer MH is an intrinsic property of Sγ3 sequence. Together, the data on ISDs and CSR indicate that the nature of the tandem repeats influences the choice of the A-NHEJ.

## Discussion

By putting Sγ3 region under the control of the known elements that regulate Sµ and by comparing the recombinational activities of Sµ and Sγ3 in the same conditions (same allele, same stimulation conditions), we provided evidence that Sγ3 acquired most of the features of Sµ. In addition to its continuous and constitutive transcription along B cell development (43) (and the present study), Sγ3 efficiently recruited AID, underwent high frequency of ISDs, and acted as a powerful donor site. Remarkably, Sγ3 acted this way despite the fact that it has a lower density of AID target motifs generally, and of the hot AGCT motif specifically (**Supplementary Table 1**). On the other hand, Sγ3 displayed a distinguishing feature, *i.e.* an increased usage of MH both in ISDs and in CSR regardless of the switch acceptor region or the stimulation condition.

It should be stressed that the A150 genetic setting has its own limitations. The fact that B cell development in A150 mice is driven by IgG3 (43) instead of IgM has already been noted. Mechanistically, it is presently unclear to what extent insertion of Eµ enhancer upstream of Sγ3 has perturbed various parameters that are important for CSR including the global architecture of the *IgH* constant region, CSRC interactions (see below), transcription of a subset of S regions and its correlation with CSR efficiency, as well as the (co-)transcriptional events and the epigenetic landscape at Sγ3 itself, which are crucial for AID recruitment and activity (7, 9). These topics clearly require further investigations.

With these caveats in mind, we found that surface expression of IgG1 and IgA was reduced in activated A150 B cells. This cannot be readily explained by defective switch transcription as Sγ1 transcript levels were only moderately reduced, while Sα (and Sε) transcript levels were normal. At the quantitative level, the precise threshold of switch transcript levels required for efficient CSR has not been determined yet. Nonetheless, the high frequency of CSR to Sγ1, as seen at the genomic level with CSR-HTGTS, suggests that the modest decrease of A150 Sγ1 transcript levels is not the critical issue.

A likely explanation stems from the lingering efficiency of Sµ as a donor site and the combinations of alleles with different rearrangement status. Indeed, switched sIg positive A150 B cells (other than IgG3) can originate from CSR events involving either Sµ or Sγ3. However, the majority of A150 alleles are in a DJ_H_ configuration and only a small fraction undergoes proximal V_H_-DJ_H_ recombination (43), of which 2/3 are in principle out-of-frame. Consequently, despite efficient recombining activity of Sµ at the genomic level, most of its recombination products lead to dead-ends at the Ig level. This is not the case when Sγ3 (located downstream of a pre-rearranged, in-frame VDJ sequence) acts as a donor site. Therefore, most of sIg positive cells likely derive from recombination events involving Sγ3 on alleles that did not undergo Sµ recombination (*i.e.* that did not delete Sγ3). Thus, the recombining activity of Sµ makes it difficult to establish a strong correlation between sIg expression and CSR events at the genomic level in A150 line. However, this issue was circumvented by using CSR-HTGTS, which provided a powerful tool to track, at the nucleotide resolution level, in allele- and orientation-independent manner, the recombination events involving both Sµ and Sγ3.

Although we cannot formally exclude a potential contribution of the PV_H_ promoter in A150 setting, acquisition of Sµ properties by Sγ3 is likely due to the proximity of Eµ enhancer. In normal B cells, Sµ is known to undergo CSR on both partially rearranged DJ_H_ alleles and fully rearranged V_H_DJ_H_ alleles (9). In A150 context, V_H_-DJ_H_ recombination is severely impaired in developing B cells (43), and only a small fraction of activated mature B cells express sIg (data not shown), but this does not prevent Sµ from acting as a powerful switch donor site as clearly shown by CSR-HTGTS. Our data therefore strongly suggest that provision of Eµ enhancer is sufficient to induce a high frequency of ISDs and to confer a robust donor function to A150 Sγ3 despite its different core repeats and the lower density of AID motifs. One possible explanation is that the ectopic Eµ enhancer ensures high levels of Sγ3 transcription, enabling efficient recruitment of AID. However, A150 Sγ3 region recruited AID as efficiently as Sµ despite comparatively lower levels of Sγ3 transcripts. On the other hand, we found comparable levels of Sγ3 transcripts in LPS-activated WT and A150 B cells. Nonetheless, AID was significantly enriched at Eµ-driven A150 Sγ3, but was only at the background level in WT Sγ3 (within the sensitivity limits of our ChIP assay). Taken together, these observations suggest that the apparent preferential targeting of Sµ by AID in activated normal B cells is not the consequence of specific properties of Sµ primary sequence such as repetitiveness or density of AID motifs, or of a higher transcriptional activity, but results, at least in part, from specific properties conferred by Eµ enhancer proximity. In this context, the proximity of Eµ enhancer can explain, at least in part, the relatively high frequency of sequential switching to Sε in normal B cells. Indeed, CSR to IgE is known to occur directly (Sμ/Sε) or sequentially (Sμ/Sγ1/Sε) [*e.g.*( (49–53)]. The presence of Eµ upstream of the hybrid Sµ/Sγ1 likely promotes the subsequent recombination to Sε. We do not infer from the above discussion that primary sequence peculiarities of S sequences have no importance. They have, in particular with respect to the mechanistic aspects of DSBs repair (see below).

CSR takes place in CSRCs and involves Cohesin-mediated loop extrusion that is impeded by Eµ enhancer and the 3’RR (11) [Reviewed in (9)], as well as the super-anchor located downstream of the *IgH* locus which focuses loop extrusion on the upstream constant region (54). Our findings could be explained by a model whereby the two Eµ enhancers and the 3’RR co-exist in a « ménage à trois » within the CSRC. Alternatively, there may be competition between the two Eµ elements such that only one lies close to the 3’RR (**Supplementary Figure 8**). The high frequency of Sµ/Sγ1 and Sγ3/Sγ1 recombination on one hand, and the low frequency of Sµ/Sγ3 recombination on the other hand, favor the view that only one Eµ enhancer lies within the CSRC at a time. Nonetheless, both enhancers may co-exist in a small fraction of the CSRCs allowing the low levels Sµ/Sγ3 synapsis. Further analyses are needed to unravel the dynamics of Sµ and Sγ3 sequences within the CSRC.

In agreement with the notion that C-NHEJ is the major repair pathway during CSR (27, 28), the vast majority of A150 B cells displayed either direct or low MH joins regardless of the switch donor site. The same holds true for ISDs. Nonetheless, there was a relatively higher MH usage by Sγ3 in the context of both ISDs and CSR irrespective of the stimulation condition. Overall, the increase was moderate (_~_2-fold) but highly reproducible and statistically significant. Based on the criterion of MH extent, this suggests that Sγ3 tends to favor the recruitment of A-NHEJ regardless of the outcome of the DNA DSBs (*i.e.* ISDs or CSR) and of the acceptor site. This bias appears therefore to be directed by Sγ3, not by sequence homology with the partner S regions. We propose the following speculative model to account for this finding. Upon B cell activation, AID initially targets the switch donor region ultimately leading to multiple and heterogeneous DNA ends that recruit C-NHEJ and A-NHEJ pathways. When the partner S region is targeted by AID, the DNA ends that did not undergo short-range repair (ISDs) at the donor site will engage in *bona fide* CSR while tethering the components of the pathway initially recruited.

The increased usage of MH by Sγ3 likely reflects the complexity of its repeats and therefore the complexity of the DNA ends generated, and potentially the kinetics of repair. For instance, by focusing on the most abundant motif, the core Sµ is virtually a multimer of AGCT(G)GGGT motifs whereas the AGCT units are relatively more distant within Sγ3 repeats. It is plausible that if AID-initiates nicks on both strands of the palindromic, overlapping (55) AGCT motifs (be it at Sµ or Sγ3), or on very close AGCT motifs (more frequently at Sµ than Sγ3), the resulting ends would require very limited resection (and/or filling), ultimately leading to C-NHEJ-mediated repair. In contrast, if AID initiates nicks at distant motifs on opposite strands (more frequent at Sγ3 than Sµ), the long overhangs would require more extensive resection (and/or filling), favoring MH search and usage and A-NHEJ-mediated repair. This is in agreement with the notion that the structure of staggered DSBs influences the mode of end processing and recruitment of A-NHEJ during CSR (56–58). Recent work strongly suggests that accumulation of DNA : RNA hybrids at S regions due to deficiency of the RNA exosome catalytic subunit, DIS3, yields longer overhangs and increased MH (59). In this regard, it is possible that A150 Sγ3 DNA : RNA hybrids are relatively more stable and processed by the RNA exosome with a slower kinetics than their Sµ counterparts.

It is arguable if increased usage of MH is promoted by DNA damage response deficiencies (39–41, 60–63) and/or by other factors. The most recent evidence shows that 53BP1- and, to a lesser extent, ATM-, H2AX- and Rif1-deficiencies significantly increase MH-mediated CSR junctions (64, 65). Our data are in line with the notion that A-NHEJ can operate in the presence of intact DNA damage response. This does not exclude the possibility of enhanced MH usage in the context of defective DNA damage response. On the other hand, RAD52 has been shown to play an important role in MH-mediated A-NHEJ during CSR, notably by facilitating a KU-independent DNA DSB repair (66). To what extent the DNA damage response and RAD52 are involved in Sγ3 related A-NHEJ in CSR are questions for future investigations. Finally, the A-NHEJ was initially thought to prevail in CSR upon C-NHEJ deficiency, and whether it is efficient in C-NHEJ-proficient cells was much debated (27, 29, 30). Our findings support the notion that A-NHEJ can operate in C-NHEJ-proficient cells (65, 67) undergoing ISDs and CSR.

## Data Availability Statement

The datasets presented in this study can be found in online repositories. The names of the repository/repositories and accession number(s) can be found below: https://www.ncbi.nlm.nih.gov/geo/, GSE174296.For FACS data / FlowRepositery ID : FR-FCM-Z3SX Access with the following link : https://flowrepository.org/id/RvFrFuSmwmwDXnmHOsLKZvnlUyMgrKRdBybKuJo4HfMcahREDfK4mNLE3OHJvSYG.

## Ethics Statement

The animal study was reviewed and approved by The Regional Ethical Committee (Accreditation N° E31555005). All experiments on mice have been carried out according to the CNRS ethical guidelines.

## Author Contributions

CO, XZ, NP, FA, and AK actively participated to the experimental design of the study. CO, XZ, NK, AK, and FA designed CSR-HTGTS and interpretation of the data. CL and F-ZB contributed to experiments. AD handled the mouse lines. All authors participated in the scientific discussion for manuscript writing, read and approved the manuscript. AK designed the project and obtained financial grants and agreement of the relevant ethic committees to perform the study. All authors contributed to the article and approved the submitted version.

## Funding

This work was supported by the Agence Nationale de la Recherche [ANR-21-CE15-0019], the Institut National du Cancer [INCA_9363, PLBIO15-134], the Fondation ARC pour la Recherche sur le Cancer [PJA 20191209515], the Ligue Contre le Cancer (Ligue Régionale : comités de l’Ex Région Midi-Pyrénées). FA is an investigator of the Howard Hughes Medical Institute. CO was a fellow of the Ministry of Higher Education & Research and recipient of a fellowship from the “Fondation pour la Recherche Médicale”. TRI- IPBS has the financial support of ITMO Cancer Aviesan (National Alliance for Life Science and Health) within the framework of Cancer Plan.

## Conflict of Interest

The authors declare that the research was conducted in the absence of any commercial or financial relationships that could be construed as a potential conflict of interest.

## Publisher’s Note

All claims expressed in this article are solely those of the authors and do not necessarily represent those of their affiliated organizations, or those of the publisher, the editors and the reviewers. Any product that may be evaluated in this article, or claim that may be made by its manufacturer, is not guaranteed or endorsed by the publisher.

## Supplementary Material

Supplementary Figure 1 FACS analysis of A150 resting splenic B cells. **(A)** The top scheme indicates the structure of the A150 allele where Iγ3 promoter was replaced by a PVH-VDJ-Eµ cassette. **(B)** CD43-negatively sorted splenic B cells were stained with anti-B220 and either anti-IgM or anti-IgG3. The vast majority (>98%) of A150 resting B cells express surface IgG3 (n = 3).

Supplementary Figure 2 Switch transcription in resting B cells. **(A)** The scheme indicates the structure of the A150 allele and µ and γ3 transcription units each derived from its proximal Eµ/Iµ enhancer/promoter, and their Sµ and Sγ3 transcripts respectively. The two sets of transcripts can easily be distinguished by using reverse primers specific of Cµ and Cγ3 respectively. The relative position of the primers used to detect spliced switch transcripts is indicated. **(B)** Quantification of Sµ transcript levels in WT and A150 resting B cells. Total RNAs were prepared from purified CD43^-^ WT and A150 B cells, reverse transcribed, and Sµ transcript levels quantified by RT-qPCR (n = 8). **(C)** Comparison of Sµ and Sγ3 transcript levels in A150 resting B cells. Quantification of switch transcript levels was as in **(B)**. Because the Cµ and Cγ3 reverse primers are different, the comparison is based on ΔCt data (n = 8).

Supplementary Figure 3 Switch transcription in activated B cells. **(A)** The scheme indicates the structure of the A150 allele and µ and γ3 transcription units each derived from its proximal Eµ/Iµ enhancer/promoter, and their Sµ and Sγ3 transcripts respectively. The two sets of transcripts can easily be distinguished by using reverse primers specific of Cµ and Cγ3 respectively. The relative position of the primers used to detect spliced switch transcripts is indicated. **(B)** Quantification of Sµ transcript levels in WT and A150 activated B cells. Total RNAs were prepared from purified CD43^-^ WT and A150 B cells at day 2 post-stimulation with anti-CD40+IL4 (left) or anti-CD40+TGFβ (right), reverse transcribed, and Sµ transcript levels quantified by RT-qPCR (n = 4). **(C)** Comparison of Sµ and Sγ3 transcript levels in activated A150 B cells. Quantification of switch transcript levels was as in **(B)**. Because the Cµ and Cγ3 reverse primers are different, the comparison is based on ΔCt data (n = 8) (n ≥ 4). **(D)** The A150 mutation differentially affects switch transcription of downstream S regions. Total RNAs were prepared from purified CD43^-^ WT and A150 B cells at day 2 post-stimulation, and the transcript levels quantified as in **(B)** (n = 4). The scheme on the bottom illustrates the downstream transcription units and indicates the relative position of the primers used to detect the spliced forms of the switch transcripts (x stands for γ1, ϵ or α). Note that due to the presence of three splice donor sites on the primary Sα transcript, the splicing reaction produces three mature transcripts. For the sake of quantification, only one mature transcript was reverse transcribed.

Supplementary Figure 4 Surface expression of IgG1 and IgA on activated B cells. CD43^-^ sorted splenic B cells with the indicated genotypes were induced to switch to IgG1 (anti-CD40+IL4), or to IgA (anti-CD40+TGFβ). At day 4.5 post-stimulation, the cells were stained with the indicated antibodies. Representative plots are shown. Anti-CD40+IL4 (WT, n=6; A150, n=7), anti-CD40+TGFβ (WT, n=3; A150, n=4). The histograms recapitulating the flow cytometry experiments are shown on the right.

Supplementary Figure 5 Switch transcription in LPS-activated B cells. **(A)** The scheme depicts the structure of WT and A150 γ3 transcription units derived from their proximal Iγ3 promoter and Eµ/Iµ enhancer/promoter, respectively, and their Sγ3 transcripts. The two sets of transcripts can easily be distinguished by using forward primers specific of Eµ and Iγ3 respectively. The relative position of the primers used to detect spliced switch transcripts is indicated. **(B)** Quantification of Sγ3 transcript levels in LPS-activated B cells. Total RNAs were prepared from purified CD43^-^ WT and A150 B cells at day 2 post-stimulation with LPS, reverse transcribed, and Sγ3 transcript levels quantified by RT-qPCR. Because the Eµ/Iµ and Iγ3 forward primers are different, the comparison is based on ΔCt data (n ≥ 8).

Supplementary Figure 6 Increased micro-homology usage in switch junctions involving Sγ3 as a donor site upon anti-CD40+IL4 stimulation. **(A, B)** MH-mediated joining was analyzed in A150 B cells stimulated with anti-CD40+IL4 for 4.5 days. MH usage from junctions with blunt and up to 3-bp MH, and >3-bp MH were plotted as percentage of total junctions involving Sµ or Sγ3 as switch donor sites and either Sγ1 **(A)** or Sϵ **(B)** as acceptor sites. The number of switch junctions and of independent mice are indicated between brackets. The p values were calculated by unpaired two-tailed t test. **(C)** Examples of switch junctions obtained with either Sµ (left panel) or Sγ3 (right panel) as donor sites and Sϵ as acceptor site. MH at switch junctions is highlighted in pale blue box.

Supplementary Figure 7 Increased micro-homology usage in switch junctions involving Sγ3 as a donor site upon anti-CD40+TGF-γ stimulation. **(A–C)** MH-mediated joining was analyzed in A150 B cells stimulated with anti-CD40+TGF-β for 4.5 days. MH usage from junctions with blunt and up to 3-bp MH, and >3-bp MH were plotted as percentage of total junctions involving Sµ or Sγ3 as switch donor sites and Sγ1 **(A)**, Sϵ **(B)**, or Sα **(C)** as acceptor sites. The number of switch junctions and of independent mice are indicated between brackets. The p values were calculated by unpaired two-tailed t test. **(D)** Examples of switch junctions obtained with either Sµ (left panel) or Sγ3 (right panel) as donor sites and Sα as acceptor site. MH at switch junctions is highlighted in pale blue box.

Supplementary Figure 8 A speculative model for CSR events in A150 in the context of CSRC. In this model, long-range interactions between Eµ enhancers and the 3’RR generate a CSRC. The three elements are Cohesin-binding sites (blue ring) and act as dynamic impediments to Cohesin-mediated loop extrusion bringing S sequences into proximity. In the two major fractions of CSRCs, loop extrusion would juxtapose Sγ1 to either Sµ or Sγ3, enabling Sµ/Sγ1 and Sγ3/Sγ1 CSR respectively. In a minor fraction of CSRCs, loop extrusion would juxtapose Sγ3 to Sµ enabling inversional CSR. The super-anchor downstream of the 3’RR is not shown.
