# Supplementary material for: Switch Tandem Repeats Influence the Choice of the Alternative End-Joining Pathway in Immunoglobulin Class Switch Recombination
Source: Front Immunol. 2022 May 16;13:870933. doi: 10.3389/fimmu.2022.870933 (PMC9149575; doi:10.3389/fimmu.2022.870933)
Supplement: Supplementary file 1 [file DataSheet_2.pdf]

Supplementary table 1

AID target motifs at Sμ and Sγ3 regions

Various nomenclatures are used for AID target motifs, which lead to different numbers of the motifs at Sμ and Sγ3 regions. Therefore, we report the numbers according to each nomenclature by highlighting the hot agct motif. It is difficult to delineate precisely the borders of the core S sequence. Therefore, the numbering is based on the whole intron sequence.

R : A or G **W : A or T** **Y : C or T** **D : A or G or T**

WRCW

S region (129Sv)	Intron size (bp)	agca	agct	aaca	aact	tgca	tgct	taca	tact	Total WRCW	WRCW/100 bp	agct/100 bp
Sμ	5625	11	432	7	29	4	15	9	13	520	9.24	7.68
Sγ3	4918	30	108	13	19	11	11	20	4	216	4.39	2.19

WGCW

S region (129Sv)	Intron size (bp)	agca	agct	tgca	tgct	Total WGCW	WGCW/100 bp	agct/100 bp
Sμ	5625	11	432	4	15	462	8.21	7.68
Sγ3	4918	30	108	11	11	160	3.25	2.19

RGYW

S region (129Sv)	Intron size (bp)	agca	agct	agta	agtt	ggca	ggct	ggta	ggtt	Total RGYW	RGYW/100 bp	agct/100 bp
Sμ	5625	11	432	11	15	7	60	10	14	560	9.95	7.68
Sγ3	4918	30	108	19	14	46	44	39	30	330	6.71	2.19

DGYW

S region (129Sv)	Intron size (bp)	agca	agct	agta	agtt	ggca	ggct	ggta	ggtt	tgca	tgct	tgta	tgtt	Total DGYW	DGYW /100 bp	agct/ 100 bp
Sμ	5625	11	432	11	15	7	60	10	14	4	15	12	9	600	10.66	7.68
Sγ3	4918	30	108	19	14	46	44	39	30	11	11	13	4	369	7.5	2.19

Supplementary table 2

Primers used in this study

CSR-HTGTS

ZXF840_5'Sm2-bio	/5Biosg/GAAAAATGTTGCCTGTTAACCAA
ZXF841_5'Sm2-red	ACACTCTTTCCCTACACGACGCTCTTCCGATCT tgtgggtttgaattttgaatct
ZXF888_5'Sm2_red	ACACTCTTTCCCTACACGACGCTCTTCCGATCT GGTC tgtgggtttgaattttgaatct
ZXF889_5'Sm2_red	ACACTCTTTCCCTACACGACGCTCTTCCGATCT CAGC tgtgggtttgaattttgaatct
ZXF890_5'Sm2_red	ACACTCTTTCCCTACACGACGCTCTTCCGATCT TGTA tgtgggtttgaattttgaatct
ZXF891_5'Sm2_red	ACACTCTTTCCCTACACGACGCTCTTCCGATCT GAAT tgtgggtttgaattttgaatct
ZXF892_5'Sm2_red	ACACTCTTTCCCTACACGACGCTCTTCCGATCT TACA tgtgggtttgaattttgaatct
ZXF893_5'Sm2_red	ACACTCTTTCCCTACACGACGCTCTTCCGATCT ACAT tgtgggtttgaattttgaatct
ZXF842_5'Sg3-bio	/5Biosg/TGAGAGGAACTGAGGCACCTA
ZXF843_5'Sg3-red	ACACTCTTTCCCTACACGACGCTCTTCCGATCT atgtgggagctggtgtagct
ZXF894_5'Sg3-red	ACACTCTTTCCCTACACGACGCTCTTCCGATCT GGTC atgtgggagctggtgtagct
ZXF895_5'Sg3-red	ACACTCTTTCCCTACACGACGCTCTTCCGATCT CAGC atgtgggagctggtgtagct
ZXF896_5'Sg3-red	ACACTCTTTCCCTACACGACGCTCTTCCGATCT TGTA atgtgggagctggtgtagct
ZXF897_5'Sg3-red	ACACTCTTTCCCTACACGACGCTCTTCCGATCT GAAT atgtgggagctggtgtagct
ZXF898_5'Sg3-red	ACACTCTTTCCCTACACGACGCTCTTCCGATCT TACA atgtgggagctggtgtagct
ZXF899_5'Sg3-red	ACACTCTTTCCCTACACGACGCTCTTCCGATCT ACAT atgtgggagctggtgtagct

ChIP

Sμ-1	5’Sμ3F	CAGGTCGGCTGGACTAACTCTC	63°C
	SμH3R1	GGCTCTCAACCTTGTTCCCTTA	
Sμ-2	Sμ-1 Fw	TAAAATGCGCTAAACTGAGGTGATTACT	63°C
	Sμ-1 Rev	CATCTCAGCTCAGAACAGTCCAGTG	
Sγ3-1	Sg3F1	CTGGGTCAGGGTAGGAGGTAAT	63°C
	Sg3R1	CCTCTGTAACTGCTCCATCTGTCC	
Sγ3-2	Sg3F1N	GGTGGGTGGGGTTGTGAGG	63°C
	Sg3R1N	CCTCATACTCCCTCCTACCCCAG	
