# Supplementary material for: Switch Tandem Repeats Influence the Choice of the Alternative End-Joining Pathway in Immunoglobulin Class Switch Recombination
Source: Front Immunol. 2022 May 16;13:870933. doi: 10.3389/fimmu.2022.870933 (PMC9149575; doi:10.3389/fimmu.2022.870933)
Supplement: Supplementary Figure 1 — FACS analysis of A150 resting splenic B cells. (A) The top scheme indicates the structure of the A150 allele where Iγ3 promoter was replaced by a PVH-VDJ-Eµ cassette. (B) CD43-negatively sorted splenic B cells were stained with anti-B220 and either anti-IgM or anti-IgG3. The vast majority (>98%) of A150 resting B cells express surface IgG3 (n = 3). [file DataSheet_1.pdf]

Suppl. Fig. 1

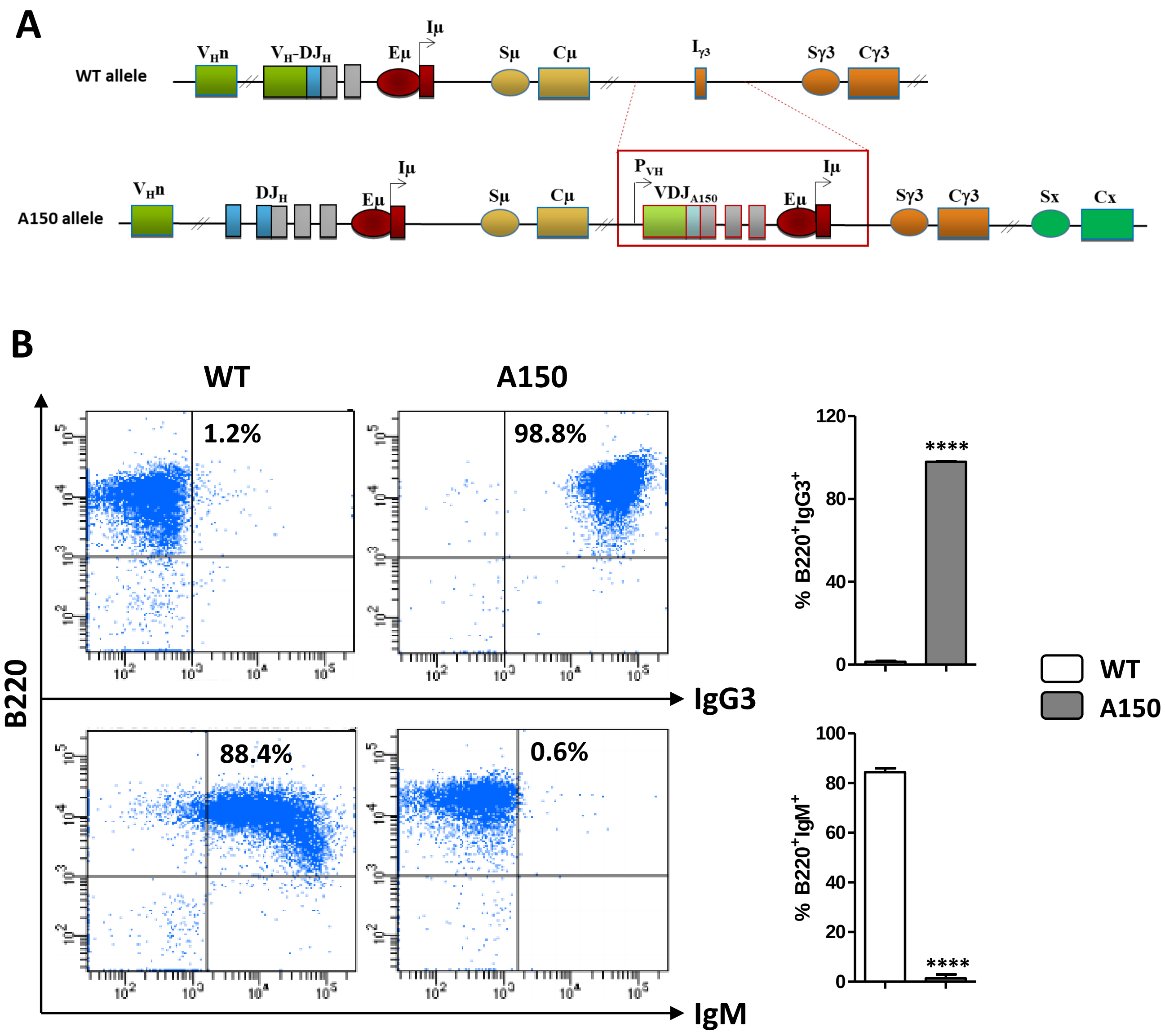

A
WT allele
A150 allele
B
WT
A150
1.2%
98.8%
88.4%
0.6%
B220
IgG3
IgM
% B220+IgG3+
% B220+IgM+
****
WT
A150

Suppl. Fig. 2

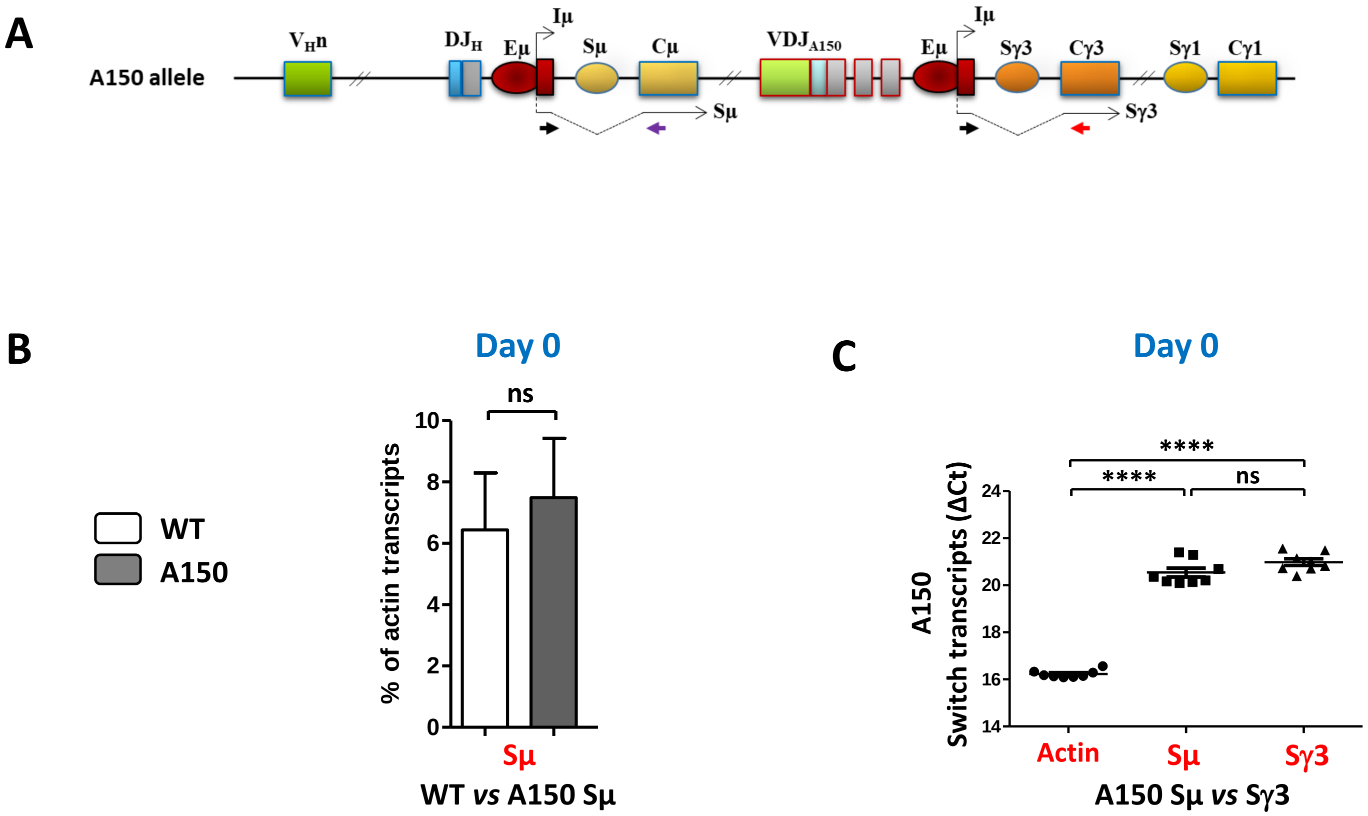
A
A150 allele
VHn
DJH
Eμ
Iμ
Sμ
Cμ
VDJA150
Sγ3
Cγ3
Sγ1
Cγ1
B
WT
A150
Day 0
ns
% of actin transcripts
0
2
4
6
8
10
Sμ
WT vs A150 Sμ
C
Day 0
****
****
ns
A150
Switch transcripts (ΔCt)
14
16
18
20
22
24
Actin
Sμ
Sγ3
A150 Sμ vs Sγ3

Suppl. Fig. 3

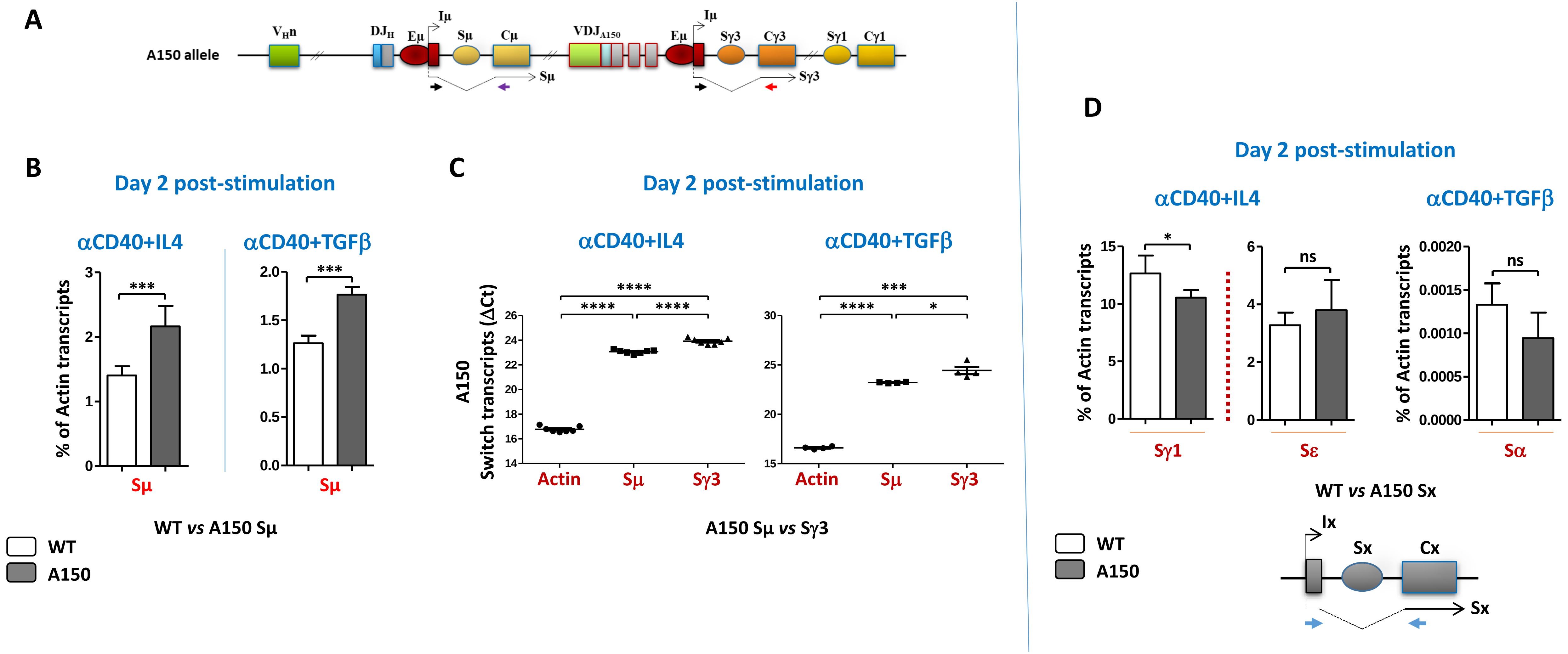
A
A150 allele
VHn
DJH
Eμ
Iμ
Sμ
Cμ
VDJA150
Sγ3
Cγ3
Sγ1
Cγ1
B
Day 2 post-stimulation
αCD40+IL4
αCD40+TGFβ
% of Actin transcripts
***
Sμ
WT vs A150 Sμ
WT
A150
C
Day 2 post-stimulation
αCD40+IL4
αCD40+TGFβ
A150
Switch transcripts (ΔCt)
****
***
*
Actin
Sμ
Sγ3
A150 Sμ vs Sγ3
D
Day 2 post-stimulation
αCD40+IL4
αCD40+TGFβ
% of Actin transcripts
*
ns
Sγ1
Sε
Sα
WT vs A150 Sx
WT
A150
Ix
Sx
Cx

Suppl. Fig. 4

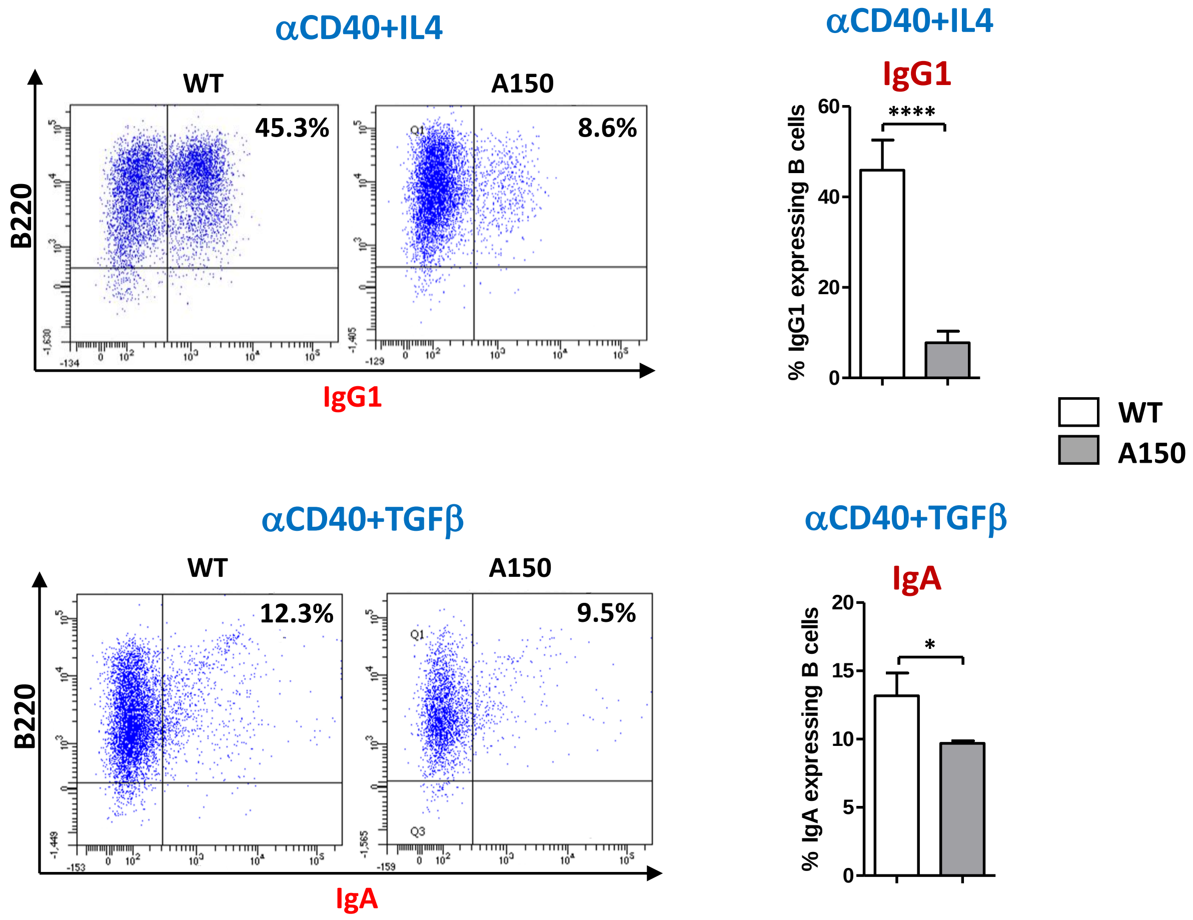
αCD40+IL4
WT
A150
45.3%
8.6%
B220
IgG1
αCD40+IL4
IgG1
% IgG1 expressing B cells
****
60
40
20
0
WT
A150
αCD40+TGFβ
WT
A150
12.3%
9.5%
B220
IgA
αCD40+TGFβ
IgA
% IgA expressing B cells
*
20
15
10
5
0

Suppl. Fig. 5

A

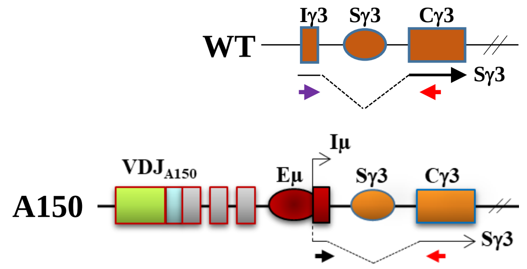

B

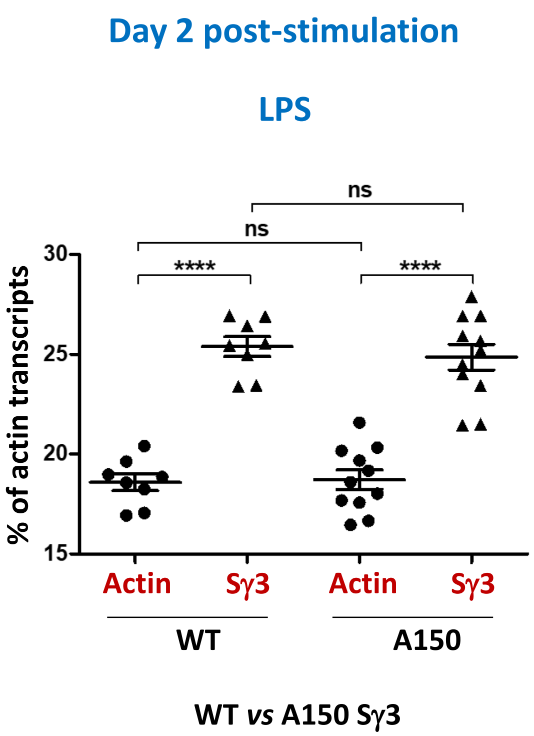

Suppl. Fig. 6

αCD40+IL4 stimulation

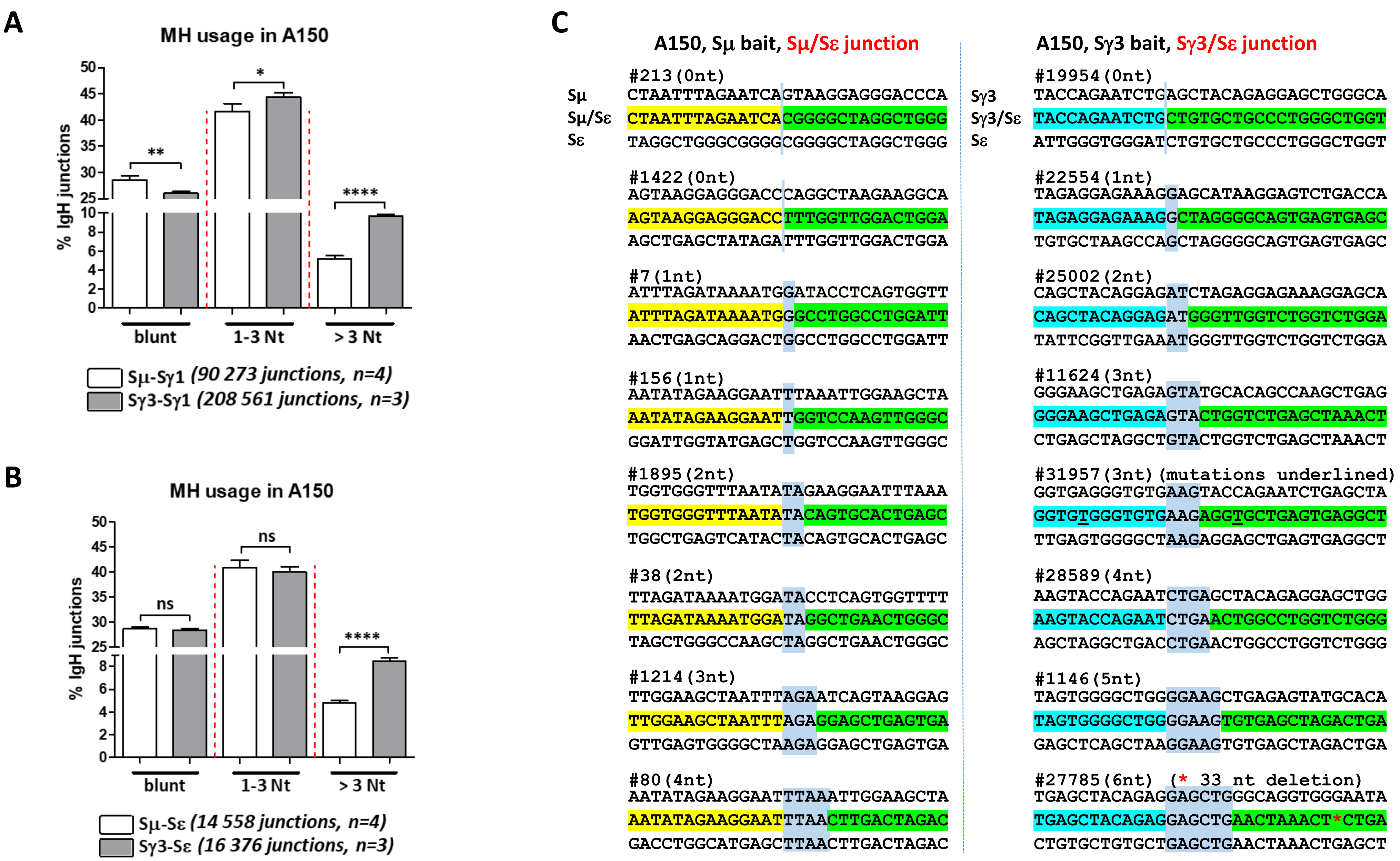

Suppl. Fig. 7

αCD40+TGFβ stimulation

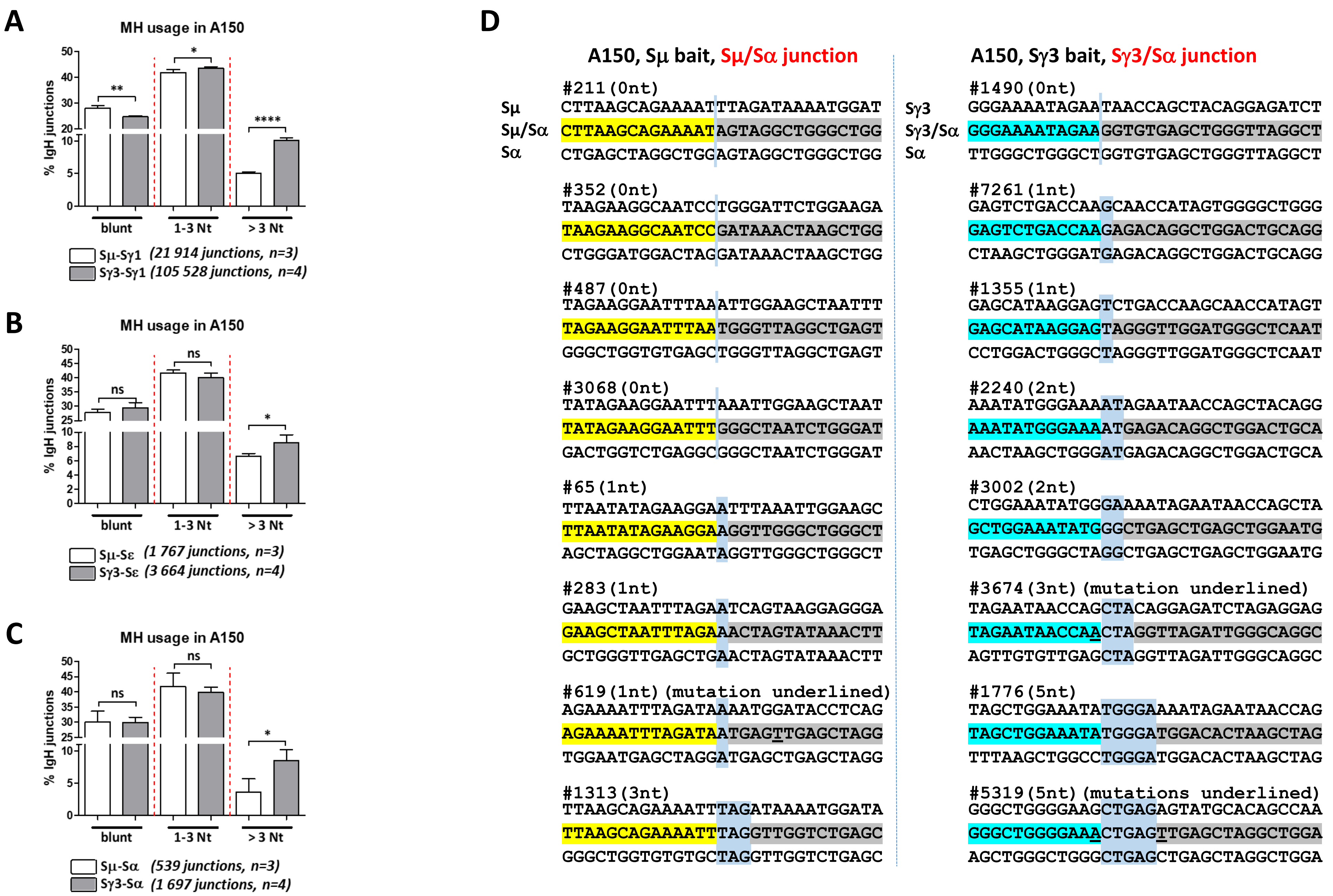

Suppl. Fig. 8

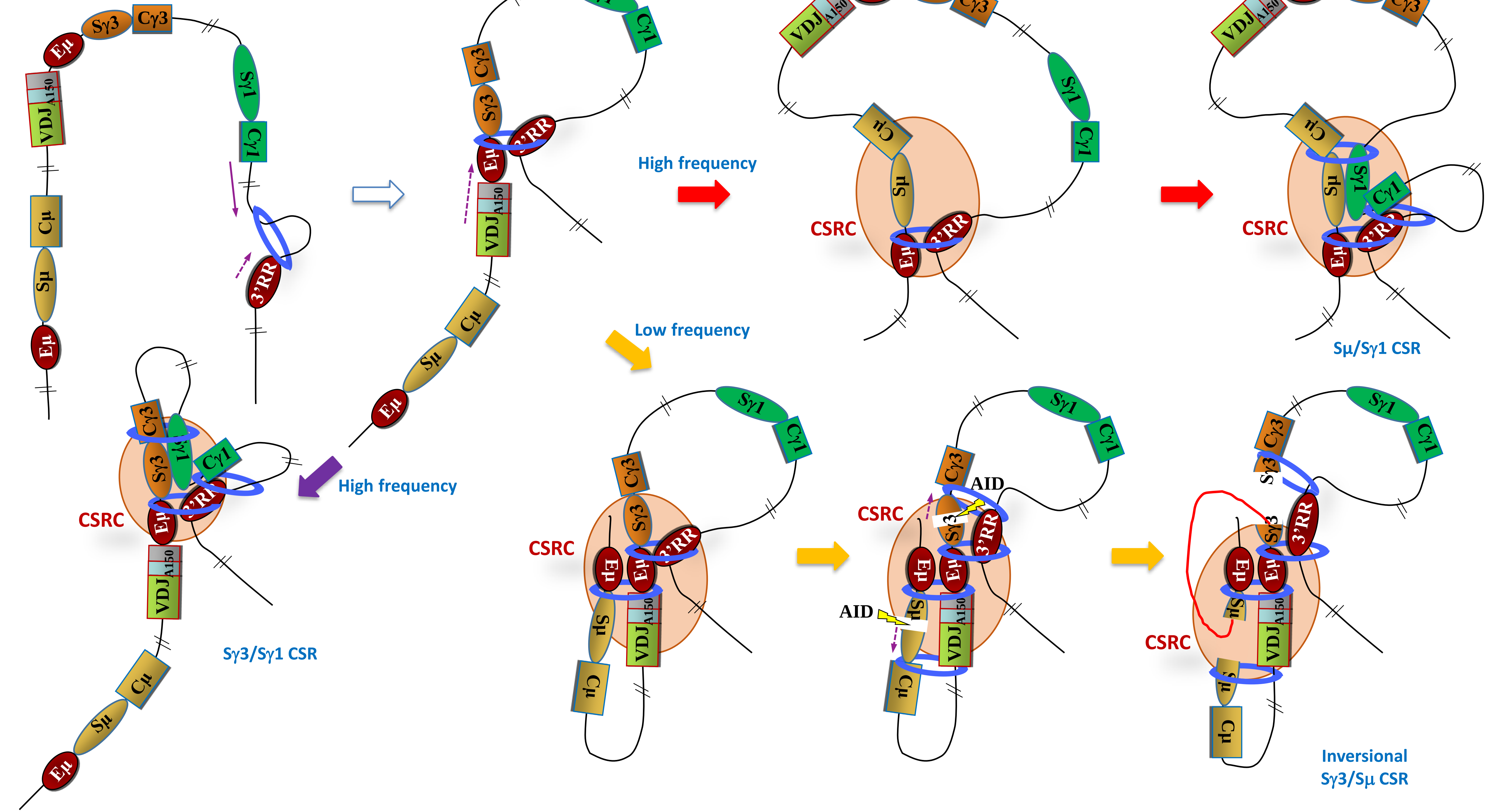

High frequency
Low frequency
High frequency
CSRC
AID
Sμ/Sγ1 CSR
Sγ3/Sγ1 CSR
Inversional
Sγ3/Sμ CSR
